# Supplementary material for: Crawling and Gliding: A Computational Model for Shape-Driven Cell Migration
Source: PLoS Comput Biol. 2015 Oct 21;11(10):e1004280. doi: 10.1371/journal.pcbi.1004280 (PMC4619082; doi:10.1371/journal.pcbi.1004280)
Supplement: S1 Code — (ZIP) [file pcbi.1004280.s012.zip › release/tst/doc/html/x11graph_8h.html]

Tissue Simulation Toolkit: x11graph.h File Reference


Tissue Simulation Toolkit
 0.1.4.1


- Main Page
- Namespaces
- Classes
- Files

- File List
- File Members

Classes |
Macros |
Typedefs

x11graph.h File Reference

`#include <X11/Xlib.h>`  
`#include <X11/Xutil.h>`  
`#include <X11/keysym.h>`  
`#include <X11/cursorfont.h>`  
`#include "graph.h"`

Include dependency graph for x11graph.h:

This graph shows which files directly or indirectly include this file:

Go to the source code of this file.

|  |  |
| --- | --- |
| Classes | |
| struct | li |
|  | |
| struct | co |
|  | |
| class | X11Graphics |
|  | X-Windows implementation of Graphics interface. More... |
|  | |

|  |  |
| --- | --- |
| Macros | |
| #define | OUTFILE   "beestje.mov" |
|  | |
| #define | CFILE   "sticky.ctb" |
|  | |
| #define | VERBOSE   1 |
|  | |
| #define | RESIZE   -20 |
|  | |
| #define | MOTION   -21 |
|  | |
| #define | TIMESTEP   void X11Graphics::TimeStep(void) |
|  | |

|  |  |
| --- | --- |
| Typedefs | |
| typedef struct li | LineType |
|  | |
| typedef struct co | Coordinate |
|  | |

## Macro Definition Documentation

#define CFILE   "sticky.ctb"

#define MOTION   -21

Referenced by X11Graphics::GetXYCoo(), and X11Graphics::ReplaceBeast().

#define OUTFILE   "beestje.mov"

#define RESIZE   -20

Referenced by X11Graphics::GetXYCoo().

#define TIMESTEP   void X11Graphics::TimeStep(void)

#define VERBOSE   1

## Typedef Documentation

typedef struct co Coordinate

typedef struct li LineType


---

Generated on Thu Aug 14 2014 22:04:01 for Tissue Simulation Toolkit by  

 1.8.6
